# Pediatric Asthma in a Universally Insured Military Population

**DOI:** 10.1001/jamanetworkopen.2025.56740

**Published:** 2026-01-26

**Authors:** Felicia Yeboah Denteh, Wendy Vaughan, Amanda Banaag, Hongyan Wu, Kimera A. Joseph, Tracey Pérez Koehlmoos

**Affiliations:** 1 Center for Health Services Research, Uniformed Services University of the Health Sciences, Bethesda, Maryland; 2 Henry M. Jackson Foundation for the Advancement of Military Medicine, Bethesda, Maryland; 3 Walter Reed National Military Medical Center, Children’s Center, Bethesda, Maryland

## Abstract

**Importance:**

Pediatric asthma risk is associated with socioeconomic characteristics at individual, household, and community levels. It is critical to understand how the prevalence, treatment, and outcomes of pediatric asthma differ among ethnic and racial groups in a universally insured population.

**Objective:**

To investigate racial and ethnic differences in asthma diagnoses, potentially avoidable hospitalizations (PAHs) or emergency department (ED) visits, specialist visits, and asthma-related prescriptions among Military Health System (MHS) pediatric beneficiaries.

**Design, Setting, and Participants:**

This cross-sectional study investigated the prevalence of pediatric asthma among MHS beneficiaries enrolled in TRICARE Prime during fiscal year (FY) 2023. Secondary health care and pharmacy claims data were obtained from the MHS Data Repository. Participants included TRICARE Prime–enrolled dependents, aged 2 to 17 years, who were seen in at least 1 inpatient or outpatient setting during FY 2023.

**Exposures:**

Race and ethnicity, derived from sponsor demographics, was the primary variable of interest. Controlling variables included sex, sponsor marital status, number of siblings, health care setting, region, and sponsor rank.

**Main Outcomes and Measures:**

Outcomes of interest were asthma prevalence and asthma-related outcomes (PAHs, ED visits, specialist visits, and prescriptions). Multiple logistic regressions, with 95% CIs, were used to estimate outcomes of interest within each age group and among all ages. Odds ratios (ORs) for asthma diagnosis across both age group and race and ethnicity categories were assessed among the full pediatric cohort.

**Results:**

Among the cohort of 950 896 children (largest age group, 428 212 children [45.0%] aged 11-17 years; 483 988 [50.9%] male children; 24 150 [2.5%] Asian children; 157 709 [16.6%] Black children, 166 253 [17.5%] Hispanic children, 33 032 [3.5%] Native Hawaiian or Pacific Islander children, 526 324 [55.4%] White children, and 43 428 [4.6%] children of other races), 31 288 (3.3%) (18 130 boys [1.9%]) met the asthma diagnosis definition. Greater prevalence was found in older age groups, male children, and dependents with 1 or 2 siblings. Black children had the highest odds of diagnosis for all ages (OR, 1.85; 95% CI, 1.80-1.91; *P* < .001). Black (OR, 1.39; 95% CI, 1.29-1.50; *P* < .001), Hispanic (OR, 1.36; 95% CI, 1.25-1.48; *P* < .001), and Native Hawaiian or Pacific Islander (OR, 1.25; 95% CI, 1.05-1.48; *P* = .01) children had higher odds of an asthma-related ED visit. PAHs were too few (587 visits) to return statistically significant results. Within the asthma cohort, 27 625 children (88.3%) had a prescription for any asthma treatment and 19 961 (63.8%) had a prescription for an inhaled corticosteroid.

**Conclusions and Relevance:**

This cross-sectional study of asthma prevalence and outcomes found decreases in asthma diagnoses, asthma-related ED visits, and PAHs but increases in asthma treatment and inhaled corticosteroid prescriptions. The data highlighted how low-cost to no-cost health care may have led to better asthma outcomes among the MHS pediatric population.

## Key Points

**Question:**

Are there unexpected variations in the prevalence, treatment, and outcomes of pediatric asthma in the Military Health System?

**Findings:**

In this cross-sectional study of asthma prevalence and outcomes among 950 896 Military Health System pediatric dependents, 31 288 children with asthma were identified (prevalence, 3.3%). There were decreases in overall asthma-related emergency department visits and potentially avoidable hospitalizations, but increases in asthma treatments, in particular, inhaled corticosteroid prescriptions; racial and ethnic disparities were also noted.

**Meaning:**

These findings suggest that the overall number of pediatric asthma attacks, emergency department visits, and hospitalizations among Military Health System pediatric dependents has decreased, but disparities in the prevalence and outcomes persist across racial and ethnic groups despite universal insurance coverage.

## Teaser

This cross-sectional study examines racial and ethnic differences in asthma diagnoses, potentially avoidable hospitalizations or emergency department visits, specialist visits, and asthma-related prescriptions among Military Health System pediatric beneficiaries.

## Introduction

The burden of asthma remains a major public health concern affecting nearly 7.5% of the US pediatric population.^1^ Asthma surveillance data (2019-2021) from the US Centers for Disease Control and Prevention show nearly 4.6 million children younger than 18 years have asthma.^2^ With over 1.8 million asthma attacks recorded among children aged 0 to 17 years within the same period, they are among the leading causes of school absenteeism,^1^ emergency department (ED) visits, and hospitalizations among US children.^3,4,5,6,7^

The impact of asthma is profound among children living in poor communities,^8^ with several scientific reports affirming excessive asthma prevalence,^9,10^ substandard treatment, and outcomes among children of minoritized racial groups^2,11,12^ who are socioeconomically disadvantaged. Asthma tends to be more prevalent among children of Puerto Rican (21.2%) and non-Hispanic Black (14.5%) backgrounds compared with non-Hispanic White (8.2%) and Mexican American (7.5%) children.^13^ However, other studies found that minoritized children and those living in poor urban areas have a significantly higher risk of asthma-specific disease morbidity regardless of ethnicity.^5,10,11,13,14,15,16^

Previous reports^12,17^ have noted disparities in the prevalence, treatment, and outcomes of pediatric asthma owing to lack of insurance coverage and access to care, despite federal and state subsidized health insurance programs such as Medicaid and the Children’s Health Insurance Program, which offer coverage for children from low-income families.^18,19^ Lack of care coverage,^20^ inadequate access to health care, and poor quality of care, among other social determinants such as unhealthy living environments, poverty, psychosocial stressors, practices, cultural beliefs,^21^ discrimination, and racism greatly contribute to health disparities in pediatric, adult, and minoritized racial and ethnic populations.^22^

The US Military Health System (MHS) offers comprehensive insurance coverage to beneficiaries of diverse racial and ethnic populations, including nearly 2 million children.^12,23,24^ A 2010 study by Stewart et al^12^ examining differences in prevalence, treatment, and outcomes of asthma among children in the MHS reported higher rates of asthma diagnosis, asthma-related ED visits, and potentially avoidable hospitalizations (PAHs) among Black and Hispanic children compared with White children despite universal access to military treatment facilities and health insurance coverage.^12^ However, a recently published framework synthesis on racial disparities in the military health system by Koehlmoos et al^23^ suggests that universal insurance is a mitigating factor for many health care disparities. Considering this, we sought to revisit the work of Stewart et al^12^ and to investigate the current trends in pediatric asthma diagnosis, treatments, and outcomes among children in the MHS.

## Methods

For this cross-sectional study, we followed the framework of the study by Stewart et al^12^ to better facilitate comparisons of pediatric asthma prevalence and asthma-related outcomes between the 2 study periods. Definitions, statistical analysis, and model construction were pulled from the previous study unless otherwise specified. We followed the Strengthening the Reporting of Observational Studies in Epidemiology (STROBE) reporting guidelines for cross-sectional studies. Ethics approval for this research was received from the Uniformed Services University of the Health Sciences and was found exempt for review and the need for informed consent by the institutional review board of the Uniformed Services University of the Health Sciences.

### Data Source and Study Population

**Figure 1.**
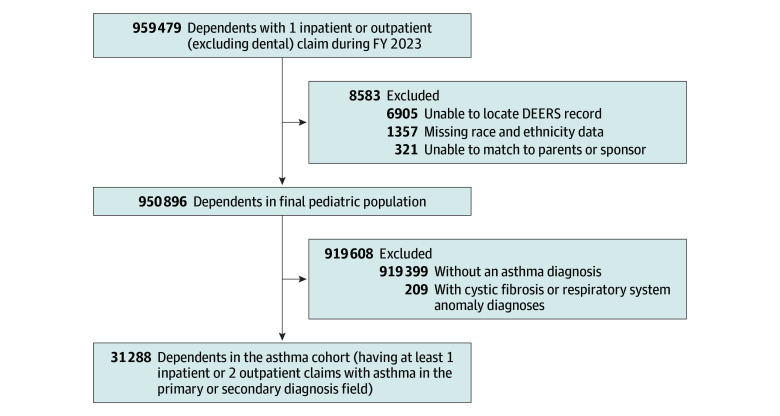
Patient Enrollment Flowchart Diagnostic codes are listed in eTable 1 in Supplement 1. DEERS indicates Defense Enrollment Eligibility Reporting System; FY, fiscal year.

This study used secondary health care and pharmacy claims data from the MHS Data Repository, a central data repository for all health care delivered to MHS beneficiaries at military treatment facilities (direct care) or civilian fee-for-service facilities (private-sector care) through the Department of War’s insurance product, TRICARE. A cross-sectional study was conducted on all TRICARE Prime enrolled pediatric beneficiaries, aged 2 to 17 years during fiscal year (FY) 2023 (October 1, 2022, to September 30, 2023). The pediatric cohort included children with at least 1 inpatient or outpatient claim during FY 2023; 1357 children with missing race and ethnicity data, 6905 children with claims that could not be matched back to the TRICARE Defense Enrollment Eligibility Reporting System, and 321 children who could not be matched to a sponsor record were excluded from the pediatric cohort (for an exclusion total of 8583). For the purposes of this study, an asthma diagnosis was defined as having at least 1 inpatient or 2 outpatient claims with an asthma diagnosis (*International Statistical Classification of Diseases and Related Health Problems, Tenth Revision [ICD-10]* diagnostic codes including J45*) in the primary or secondary diagnosis field during FY 2023. Any child with a diagnosis of cystic fibrosis, chronic respiratory disease originating in the perinatal period, or anomalies of the respiratory system during the study period were excluded from the asthma cohort (209 children) (Figure 1). *ICD-10* diagnostic codes used for inclusion and exclusion can be found in eTable 1 in Supplement 1.

### Measures

As with Stewart et al,^12^ variables were created for PAHs, ED visits, specialist visits, inhaled corticosteroids (ICS) prescriptions, and other prescription medications for the treatment and management of asthma. PAHs were defined by an admission with asthma as the primary diagnosis,^14^ while specialist visits included health care encounters with allergy clinics, pulmonary disease clinics, respiratory therapists, and ear, nose, and throat specialists. Asthma-related ED visits were defined as an ED visit with asthma as the primary diagnosis but excluded any asthma-related visits that also had additional reports of injury or accident (*ICD-10* codes beginning with S, T, V, W, X, or Y). Pharmacy data for filled prescriptions was used to determine asthma treatments, including ICSs, short-acting and long-acting β-agonists, leukotriene receptor antagonists, systemic oral corticosteroids, anticholinergics, long-acting muscarinic antagonists, combinations, and biologic medications. All unique nonasthma prescriptions filled during the study period were calculated and categorized (0-1, 2-4, 5-8, and ≥9 prescriptions) as a measure of comorbidity.

Demographic characteristics, including age, sex, number of siblings, sponsor’s marital status, sponsor’s rank as a proxy for socioeconomic status,^25^ and state of residence, were abstracted from the pediatric population’s Defense Enrollment Eligibility Reporting System record. As with the previous study,^12^ corresponding sponsor demographic data were used as a proxy to determine children’s race and ethnicity because of the limited availability of racial and ethnic information for dependents. Race and ethnicity, self-reported by the beneficiary, were reported as they appeared in the MHS Data Repository except in the following cases. In cases where the only race and ethnicity on record was Asian or Pacific Islander, the individual was included in the Asian race category. The other race classification includes the racial and ethnic identifications of other and unknown, along with the racial identification of American Indian or Alaska Native (owing to small cell counts in data stratifications). The number of siblings was determined by calculating the total number of beneficiaries classified as children, stepchildren, adopted, ward, and similar with the same sponsor. The child’s recorded US state of residence was used to determine census division. In addition, the care setting where beneficiaries received care was abstracted.

### Statistical Analysis

Descriptive analyses were conducted on demographics, military-related characteristics, and outcomes by race and ethnicity. Two-sided χ^2^ tests were used to compare the prevalence of asthma diagnosis across race and ethnic groups among the pediatric population, along with asthma-related outcomes and prescriptions among the asthma cohort. Multiple logistic regressions, with 95% CIs, were used to estimate outcomes of interest within each age group and among all ages. Odds ratios (ORs) for asthma diagnosis across both age group and race and ethnicity categories were assessed among the full pediatric cohort, while controlling for demographic and military characteristics (sex, number of siblings, health care setting used, region of residence, sponsor marital status, and sponsor rank). ORs for asthma-related outcomes (PAH, ED visit, specialist visit, any asthma prescription, any ICS prescription) across both age groups and race and ethnicity categories were estimated from the asthma cohort. The asthma-related outcomes models were controlled for sex, number of siblings, health care setting used, region of residence, number of unique nonasthma prescriptions filled during the study period, sponsor marital status, and sponsor rank. The model for ED visits was additionally controlled for specialist visits, any asthma prescription, and any ICS prescription. All analyses were conducted using SAS statistical software version 9.4 (SAS Institute), and statistical significance was set a priori to α = .05.

## Results

Among our pediatric cohort of 950 896 children (largest age group, 428 212 children [45.0%] aged 11-17 years; 483 988 [50.9%] male children; 24 150 [2.5%] Asian children; 157 709 [16.6%] Black children, 166 253 [17.5%] Hispanic children, 33 032 [3.5%] Native Hawaiian or Pacific Islander children, 526 324 [55.4%] White children, and 43 428 [4.6%] children of other races), we identified 31 288 children with asthma, for a prevalence of 3.3% for FY 2023 (Table 1). We found lower asthma prevalence among children aged 2 to 4 years (5080 children [0.5%]) compared with the other age groups of 5 to 10 years (13 280 children [1.4%]) and 11 to 17 years (12 928 children [1.4%]). Male children had a higher prevalence (18 130 children [1.9%]) than female children (13 158 children [1.4%]). Higher prevalence was found among children with 1 or 2 siblings (19 002 children [2.0%]) compared with children with 3 or more siblings (8803 children [0.9%]) and no or an unknown number of siblings (3483 children [0.4%]). Children with a married sponsor (27 985 children [2.9%]) had a higher prevalence than those with a nonmarried sponsor (3303 children [0.4%]). Children with a sponsor in the enlisted ranks had a higher prevalence than those with a sponsor with an officer rank (junior enlisted, 2261 children [0.2%]; senior enlisted, 22 482 children [2.4%]; junior officer, 3382 children [0.4%]; and senior officer, 1900 children [0.2%]).

**Table 1.** Asthma Prevalence Across Demographic and Military Characteristics by Race and Ethnicity in the Full Pediatric Cohort^a^

Characteristic	Children, No. (%)						
	Total (N = 950 896)	Asian (n = 24 150)	Black (n = 157 709)	Hispanic (n = 166 253)	Native Hawaiian or Pacific Islander (n = 33 032)	White (n = 526 324)	Other (n = 43 428)^b^
Age group, y							
All	31 288 (3.3)	763 (3.2)	7848 (5.0)	6023 (3.6)	984 (3.0)	14 349 (2.7)	1321 (3.0)
2-4	5080 (0.5)	97 (0.4)	1197 (0.8)	1101 (0.7)	192 (0.6)	2314 (0.4)	179 (0.4)
5-10	13 280 (1.4)	322 (1.3)	3247 (2.1)	2782 (1.7)	485 (1.5)	5893 (1.1)	551 (1.3)
11-17	12 928 (1.4)	344 (1.4)	3404 (2.2)	2140 (1.3)	307 (0.9)	6142 (1.2)	591 (1.4)
Sex							
Male	18 130 (1.9)	471 (2.0)	4615 (2.9)	3553 (2.1)	567 (1.7)	8192 (1.6)	732 (1.7)
Female	13 158 (1.4)	292 (1.2)	3233 (2.1)	2470 (1.5)	417 (1.3)	6157 (1.2)	589 (1.4)
No. of siblings							
0 or unknown	3483 (0.4)	103 (0.4)	1046 (0.7)	736 (0.4)	118 (0.4)	1343 (0.3)	137 (0.3)
1-2	19 002 (2.0)	498 (2.1)	4201 (2.7)	3773 (2.3)	633 (1.9)	9146 (1.7)	751 (1.7)
≥3	8803 (0.9)	162 (0.7)	2601 (1.7)	1514 (0.9)	233 (0.7)	3860 (0.7)	433 (1.0)
Sponsor marital status							
Married	27 985 (2.9)	706 (2.9)	6416 (4.1)	5410 (3.3)	876 (2.7)	13 402 (2.6)	1175 (2.7)
Single	3303 (0.4)	57 (0.2)	1432 (0.9)	613 (0.4)	108 (0.3)	947 (0.2)	146 (0.3)
Sponsor rank group^c^							
Junior enlisted	2261 (0.2)	71 (0.3)	692 (0.4)	569 (0.3)	44 (0.1)	824 (0.2)	61 (0.1)
Senior enlisted	22 482 (2.4)	564 (2.3)	6053 (3.8)	4547 (2.7)	708 (2.1)	9673 (1.8)	937 (2.2)
Junior officer	3382 (0.4)	74 (0.3)	575 (0.4)	520 (0.3)	132 (0.4)	1910 (0.4)	171 (0.4)
Senior officer	1900 (0.2)	43 (0.2)	241 (0.1)	161 (0.1)	59 (0.2)	1299 (0.3)	97 (0.2)
Health care sector							
Direct care only	1447 (0.2)	47 (0.2)	390 (0.3)	326 (0.2)	55 (0.2)	531 (0.1)	98 (0.2)
Private sector only	14 620 (1.5)	401 (1.7)	3419 (2.2)	2645 (1.6)	299 (0.9)	7236 (1.4)	620 (1.4)
Both	15 221 (1.6)	315 (1.3)	4039 (2.6)	3052 (1.8)	630 (1.9)	6582 (1.3)	603 (1.4)

^a^ For the purposes of this study, an asthma diagnosis is defined as having at least 1 inpatient or 2 outpatient claims with asthma in the primary or secondary field during fiscal year 2023.

^b^ Includes racial identifications of other, unknown, and American Indian or Alaska Native.

^c^ Other and unknown rank groups are excluded.

**Figure 2.**
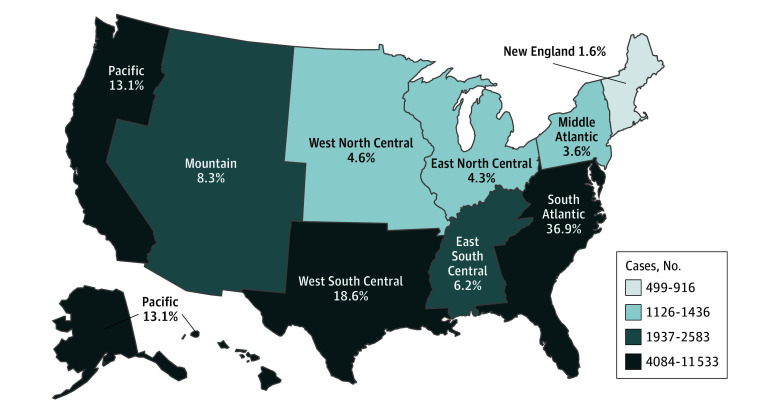
Map of Asthma Prevalence Among Military Health System Pediatric Dependents by Census Division For the purposes of this study, an asthma diagnosis is defined as having at least 1 inpatient or 2 outpatient claims with asthma in the primary or secondary diagnosis field during fiscal year 2023. Census division is determined by state of residence during the study period.

Asian (334 of 763 children [45.1%]), Black (3404 of 7848 children [43.4%]), and White (6142 of 14 349 children [42.8%]) children with asthma mostly received diagnoses between ages 11 and 17 years. Among the asthma cohort, more Asian children (401 of 763 children [52.6%]), White children (7236 of 14 349 children [50.4%]), and children with the racial and ethnic grouping of other (620 of 1321 children [46.9%]) were more likely to utilize only the private sector during the study period. The geographic distribution of children with asthma by census division can be seen in Figure 2. Distribution of the demographic characteristics for the entire pediatric population and the asthma cohort can be found in eTables 2 and 3, respectively, in Supplement 1.

Table 2 describes the distribution of outcomes and treatments across the racial and ethnic categories among the asthma cohort. During the study period, 12 629 children (40.4%) of the asthma cohort were prescribed 2 to 4 nonasthma drugs, 4614 children (14.8%) had an asthma-related ED visit, 1101 children (3.5%) saw a specialist, 27 625 children (88.3%) were prescribed any asthma drug, and 19 961 children (63.8%) were prescribed any ICS. Black children had the highest percentage of asthma-related ED visits (1365 of 7848 children [17.4%]), followed by Hispanic children (1016 of 6023 children [16.9%]) and Native Hawaiian or Pacific Islander children (163 of 984 children [16.6%]). White children had the lowest percentage of asthma-related ED visits (1766 of 14 349 children [12.3%]). Native Hawaiian or Pacific Islander (48 of 984 children [4.9%]) and Black (331 of 7848 children [4.2%]) children had the highest proportion of specialist visits. PAHs were omitted from Table 2 and Table 3 because of small numbers (587 visits) and nonsignificant results.

**Table 2.** Asthma Outcomes and Treatments by Race and Ethnicity in the Pediatric Asthma Cohort^a^

Outcome and treatments^b^	Children, No. (%)						
	Total (N = 31 288 [3.3%])	Asian (n = 763 [2.4%])	Black (n = 7848 [25.1%])	Hispanic (n = 6023 [19.3%])	Native Hawaiian or Pacific Islander (n = 984 [3.1%])	White (n = 14 349 [45.9%])	Other (n = 1321 [4.2%])^c^
No. of unique nonasthma drugs prescribed							
0-1	8195 (26.2)	220 (28.8)	2226 (28.7)	1529 (25.4)	245 (24.9)	3625 (25.3)	350 (26.5)
2-4	12 629 (40.4)	295 (38.7)	3134 (39.9)	2401 (39.9)	407 (41.4)	5848 (40.8)	544 (41.2)
5-8	7623 (24.4)	183 (24.0)	1844 (23.5)	1545 (25.7)	258 (26.2)	3482 (24.3)	311 (23.5)
≥9	284 (0.9)	65 (8.5)	644 (8.2)	548 (9.1)	74 (7.5)	1394 (9.7)	116 (8.8)
Outcomes							
Asthma-related emergency department visit^d^	4614 (14.8)	114 (14.9)	1365 (17.4)	1016 (16.9)	163 (16.6)	1766 (12.3)	190 (14.4)
Direct care only	213 (0.7)	NR^e^	63 (0.8)	64 (1.1)	NR^e^	56 (0.4)	17 (1.3)
Private sector only	1689 (5.4)	NR^e^	486 (6.2)	349 (5.8)	NR^e^	695 (4.8)	74 (5.6)
Both	2712 (8.7)	62 (8.1)	816 (10.4)	603 (10.0)	117 (11.9)	1015 (7.1)	99 (7.5)
Treatments							
Any specialist visit for asthma^f^	1101 (3.5)	13 (1.7)	331 (4.2)	206 (3.4)	48 (4.9)	461 (3.2)	42 (3.2)
Prescription drug use							
Any asthma drug	27 625 (88.3)	673 (88.2)	6880 (87.7)	5353 (88.9)	888 (90.2)	12 645 (88.1)	1186 (89.8)
Any inhaled corticosteroid	19 961 (63.8)	462 (60.6)	4999 (63.7)	3827 (63.5)	662 (67.3)	9128 (63.6)	883 (66.8)

Abbreviation: NR not reported.

^a^ Potentially avoidable hospitalization data are not shown owing to small numbers and nonsignificant results (n = 587).

^b^ Injuries and accidents are excluded.

^c^ Includes racial identifications of other, unknown, and American Indian or Alaska Native.

^d^ Determined by asthma as primary diagnosis.

^e^ Indicates cell suppression due to small numbers. To prevent back calculation, the next lowest count in the column was also suppressed.

^f^ Includes allergy clinic, pulmonary disease clinic, respiratory therapy, and ear, nose, and throat clinic.

**Table 3.** ORs by Age for Diagnosed Pediatric Asthma, Asthma-Related Outcomes, and Treatments^a^

Model	Age 2-4 y		Age 5-10 y		Age 11-17 y		All ages	
	OR (95% CI)	*P* value	OR (95% CI)	*P* value	OR (95% CI)	*P* value	OR (95% CI)	*P* value
Full analytic sample, No. of children^b^	170 915		351 769		428 212		950 896	
Asian (n = 24 150)	1.10 (0.89-1.35)	.38	1.34 (1.19-1.50)	<.001	1.22 (1.09-1.37)	<.001	1.25 (1.16-1.34)	<.001
Black (n = 157 709)	1.65 (1.53-1.78)	<.001	1.92 (1.84-2.01)	<.001	1.88 (1.80-1.96)	<.001	1.85 (1.80-1.91)	<.001
Hispanic (n = 166 253)	1.33 (1.24-1.43)	<.001	1.45 (1.39-1.52)	<.001	1.23 (1.17-1.30)	<.001	1.35 (1.31-1.39)	<.001
Native Hawaiian or Pacific Islander (n = 33 032)	1.09 (0.94-1.27)	.25	1.18 (1.07-1.30)	<.001	1.10 (0.98-1.24)	.10	1.15 (1.08-1.23)	<.001
White	1 [Reference]	NA	1 [Reference]	NA	1 [Reference]	NA	1 [Reference]	NA
Other (n = 43 428)^c^	1.13 (0.97-1.32)	.12	1.25 (1.14-1.36)	<.001	1.16 (1.07-1.27)	<.001	1.19 (1.12-1.26)	<.001
Only children with asthma diagnosis, No.	5080		13 280		12 928		31 288	
Asthma-related emergency department visit^d^^,^^e^								
Asian (n = 763)	1.50 (0.96-2.35)	.07	1.14 (0.84-1.55)	.40	1.12 (0.78-1.59)	.54	1.15 (0.94-1.41)	.18
Black (n = 7848)	1.19 (1.00-1.42)	.06	1.50 (1.34-1.68)	<.001	1.43 (1.26-1.64)	<.001	1.39 (1.29-1.50)	<.001
Hispanic (n = 6023)	1.32 (1.10-1.57)	.002	1.34 (1.19-1.52)	<.001	1.34 (1.15-1.56)	<.001	1.36 (1.25-1.48)	<.001
Native Hawaiian or Pacific Islander (n = 984)	1.15 (0.80-1.64)	.46	1.10 (0.86-1.40)	.44	1.41 (1.02-1.96)	.04	1.25 (1.05-1.48)	.01
White	1 [Reference]	NA	1 [Reference]	NA	1 [Reference]	NA	1 [Reference]	NA
Other (n = 1321)^c^	1.50 (1.05-2.13)	.02	1.14 (0.90-1.44)	.28	1.11 (0.84-1.46)	.46	1.15 (0.98-1.35)	.08
Asthma-related specialist visit^f^^,^^g^^,^^h^								
Asian (n = 763)	NA	NA	0.95 (0.51-1.77)	.87	0.66 (0.35-1.27)	.22	0.76 (0.49-1.18)	.23
Black (n = 7848)	NA	NA	1.21 (0.98-1.49)	.08	1.24 (1.02-1.50)	.03	1.17 (1.02-1.34)	.02
Hispanic (n = 6023)	NA	NA	0.93 (0.73-1.18)	.53	1.08 (0.86-1.36)	.50	0.99 (0.84-1.15)	.86
Native Hawaiian or Pacific Islander (n = 984)	NA	NA	1.12 (0.75-1.68)	.57	1.34 (0.86-2.07)	.19	1.15 (0.87-1.53)	.32
White	NA	NA	1 [Reference]	NA	1 [Reference]	NA	1 [Reference]	NA
Other (n = 1321)^c^	NA	NA	0.77 (0.47-1.26)	.29	0.90 (0.60-1.37)	.63	0.88 (0.65-1.18)	.39
Any asthma prescription^g^								
Asian (n = 763)	1.06 (0.60-1.88)	.84	1.06 (0.76-1.48)	.72	1.12 (0.88-1.42)	.37	1.07 (0.89-1.29)	.46
Black (n = 7848)	1.02 (0.83-1.25)	.88	1.09 (0.96-1.24)	.20	1.13 (1.03-1.25)	.01	1.10 (1.03-1.19)	.01
Hispanic (n = 6023)	0.90 (0.73-1.11)	.32	1.16 (1.01-1.34)	.04	1.12 (1.00-1.26)	.04	1.14 (1.05-1.23)	.002
Native Hawaiian or Pacific Islander (n = 984)	1.10 (0.70-1.73)	.68	0.96 (0.73-1.27)	.78	1.33 (0.99-1.78)	.06	1.2 (1.00-1.44)	.05
White	1 [Reference]	NA	1 [Reference]	NA	1 [Reference]	NA	1 [Reference]	NA
Other (n = 1321)^c^	1.03 (0.66-1.60)	.91	1.27 (0.97-1.67)	.09	1.33 (1.09-1.63)	.01	1.28 (1.10-1.48)	.002
Any inhaled corticosteroid^g^								
Asian (n = 763)	1.03 (0.70-1.52)	.88	0.78 (0.63-0.97)	.03	0.10 (0.83-1.20)	.97	0.92 (0.81-1.05)	.24
Black (n = 7848)	0.91 (0.80-1.05)	.20	0.10 (0.92-1.09)	.96	1.15 (1.07-1.24)	<.001	1.06 (1.01-1.12)	.03
Hispanic (n = 6023)	0.84 (0.73-0.97)	.01	1.03 (0.94-1.13)	.47	1.08 (0.99-1.18)	.07	1.04 (0.99-1.11)	.13
Native Hawaiian or Pacific Islander (n = 984)	1.16 (0.86-1.55)	.34	0.10 (0.92-1.09)	.96	1.20 (0.98-1.48)	.08	1.12 (0.99-1.27)	.07
White	1 [Reference]	NA	1 [Reference]	NA	1 [Reference]	NA	1 [Reference]	NA
Other (n = 1321)^c^	1.01 (0.76-1.35)	.94	1.14 (0.96-1.36)	.13	1.19 (1.02-1.37)	.02	1.15 (1.04-1.28)	.008

Abbreviations: NA, not applicable; OR, odds ratio.

^a^ Potentially avoidable hospitalization data are not shown owing to small numbers and nonsignificant results.

^b^ Model adjusts for sex, sponsor’s marital status, number of siblings covered by the same sponsor (0 or unknown, 1-2, or ≥3), health care setting used (direct care only, private sector only, or both), region (Northeast, Midwest, West, South, or overseas), and sponsor’s rank group (junior enlisted, senior enlisted, junior officer, senior officer, or other or unknown).

^c^ Includes racial identifications of other, unknown, and American Indian or Alaska Native.

^d^ Model adjusts for sex, sponsor’s marital status, number of siblings covered by the same sponsor (0 or unknown, 1-2, or ≥3), health care setting used (direct care only, private sector only, or both), region (Northeast, Midwest, West, South, or overseas), sponsor’s rank group (junior enlisted, senior enlisted, junior officer, senior officer, or other or unknown), any specialist visit for asthma, any prescription for asthma drugs, and prescription for inhaled corticosteroids, and the number of unique nonasthma prescriptions filed during the fiscal year (0-1, 2-4, 5-8, or ≥9).

^e^ Injuries and accidents are excluded.

^f^ Includes allergy clinic, pulmonary disease clinic, respiratory therapy, and ear, nose, and throat clinic.

^g^ Models adjust for sex, sponsor’s marital status, number of siblings covered by the same sponsor (0 or unknown, 1-2, or ≥3), health care setting used (direct care only, private sector only, or both), region (Northeast, Midwest, West, South, or overseas), sponsor’s rank group (junior enlisted, senior enlisted, junior officer, senior officer, or other or unknown), and the number of unique non-asthma prescriptions filed during the fiscal year (0-1, 2-4, 5-8, or ≥9).

^h^ The 2 to 4 year age group was removed from this analysis to ensure model convergence, for an all-ages number of 26 208.

Table 3 details the age-stratified regression results for odds of asthma among pediatric beneficiaries and the odds of selected outcomes among the asthma cohort. White children were the reference group for each model. The odds of having an asthma diagnosis for all ages was significantly higher for every race and ethnicity group compared with White children: Asian children, OR, 1.25 (95% CI, 1.16-1.34; *P* < .001); Black children, OR, 1.85 (95% CI, 1.80-1.91; *P* < .001); Hispanic children, OR, 1.35 (95% CI, 1.31-1.39; *P* < .001); and Native Hawaiian or Pacific Islander children, OR, 1.19 (95% CI, 1.12-1.26; *P* < .001). Black and Hispanic children had higher odds of asthma across each age group. For all ages, Black (OR 1.39; 95% CI, 1.29-1.50; *P* < .001), Hispanic (OR, 1.36; 95% CI, 1.25-1.48; *P* < .001), and Native Hawaiian or Pacific Islander (OR, 1.25; 95% CI, 1.05-1.48; *P* = .01) children had higher odds of an asthma-related ED visit. Black (OR, 1.10; 95% CI, 1.03-1.19; *P* = .01) and Hispanic (OR, 1.14; 95% CI, 1.05-1.23; *P* = .002) children had higher odds of having any asthma prescription for all ages.

## Discussion

In this cross-sectional study of asthma prevalence and outcomes among MHS pediatric dependents, we identified 31 288 military-connected children with asthma, a prevalence of 3.3% among our pediatric cohort. Prior research conducted by Stewart et al^12^ reported 59 266 asthmatic children with higher rates of asthma diagnosis, asthma-related ED visits, and PAHs among Black and Hispanic children compared with White children after adjusting for differences in socioeconomic status and demographic characteristics during calendar year 2007.^16^ Although we found a decrease in asthma prevalence from 7.2% in the study by Stewart et al^12^ to 3.3% in the current study, we also recorded a decrease in asthma-related hospitalizations and ED visits, in line with national trends.^26,27,28,29^ These national trends suggest that the decrease in our asthma cohort may be a byproduct of modified survey methods and implementation before and after the COVID-19 pandemic.^30^

Although Stewart et al^12^ limited their full pediatric cohort to beneficiaries who could be classified as Black non-Hispanic, Hispanic, and White non-Hispanic, we were able to include additional racial categories of Asian, Native Hawaiian or Pacific Islander, and other using sponsor race and ethnicity values as a proxy for their children. Asian, Black, Hispanic and Native Hawaiian or Pacific Islander, as well as children within the other race and ethnicity category, were significantly more likely to receive a diagnosis of asthma than their White counterparts, with Black children having the highest odds across every age group, consistent with national trends.^10,28^ In addition, our results do not appear to differ from Stewart et al^12^ and other national studies,^26,27^ indicating that the underlying disparities associated with the causes of asthma have not shifted in the years since the previous studies.

Overall, better asthma outcomes have been reported in the direct care system^12^ compared with the US private-sector care system,^26,27,31^ regardless of the similarities in trends. Although our results reflect the national trend of decreased asthma-related hospitalizations and ED visits,^26,29^ asthma-related PAHs were so few that statistical significance was not found in our analysis among our cohort. Our findings also indicate that Black, Hispanic, and Native Hawaiian or Pacific Islander children were more likely to have an asthma-related ED visit than their Asian and White peers. However, compared with the findings of Stewart et al,^12^ Black children in our cohort had lower odds of asthma-related ED visits, which may be a mitigation of the national trend, perhaps owing to no-cost urgent care and ED utilization for MHS beneficiaries. Our data indicate that even though Black children have benefited from the national trend of decreased asthma-related ED visits across all races, disparities persist among minoritized racial groups in the MHS.

A potential factor associated with the decrease in hospitalizations and ED visits seen among our population could be an increase in asthma prescriptions,^13^ including ICS. Stewart et al^12^ found that 70.9% to 76.0% of their asthma cohort had an active asthma prescription during their study period. Our study found that 88.3% of our asthma cohort had an active asthma prescription during the study period. An even greater increase was seen in ICS prescriptions (31.1%-34.7% in the study by Stewart et al^12^ vs 63.8% in the current study). This appears to differ from national trends. In 2019, the Global Initiative for Asthma made substantial changes to their recommendations for the treatment of intermittent and mild asthma. These recommendations^32^ included the use of daily low-dose to symptom-driven ICS, moving away from the previous recommendation of short-acting β2-antagonists alone. Studies^33,34^ have found low implementation of these new guidelines, with potential barriers to implementation including lack of awareness, cost, and insurance coverage. Without the barriers of cost and insurance coverage, our data may reflect better adherence to the updated Global Initiative for Asthma guidelines.

The current findings strengthen the evidence of how universal health coverage with low and no copayment in the MHS and effective asthma prescription and treatment regimens mitigate asthma control leading to reduced urgent care visits and overall health service utilization. Similarly, decent health insurance coverage and easy access to health care is deemed beneficial in the control of asthma^35^ in the general population. Other past studies identified public insurance, partial annual coverage, or a complete lack of health insurance^26,36^ as some known cost barriers that contribute to poor asthma care. Suri et al^37^ equated having health insurance to improved asthma outcomes, a relationship completely facilitated by cost barriers.

### Limitations

This study has limitations that should be mentioned. Our study included Hispanic ethnicity and additional racial categories of Asian, Native Hawaiian or Pacific Islander, and other using sponsor race and ethnicity values as a proxy for their dependents. Although this reduces the missing race and ethnicity values that we would otherwise have, this also introduces error into our study.^38^ In cases of multiracial children, adoption, and wards, race and ethnicity values may be misclassified. Another limitation of this study includes relying on coding in secondary data, which lends itself to coding errors and the potential for inaccurate prevalence estimates of asthma. Rank, used as a proxy for socioeconomic status,^25^ may not reflect always household income. In addition, our analysis does not include potential comorbidities such as body mass index, pollution, and family history. There is evidence that asthma is widely misdiagnosed.^36,39^ Pediatric asthma diagnosis is susceptible to both underdiagnosis and overdiagnosis, often influenced by coding practices and diagnostic variability. Coding systems, such as the International Classification of Primary Care, play a role in these diagnostic challenges. In the aforementioned Dutch study,^40^ many children received the International Classification of Primary Care code for asthma without confirmatory testing, highlighting how coding based on symptoms alone can contribute to overdiagnosis.

## Conclusions

In this cross-sectional study of asthma prevalence and outcomes among MHS pediatric dependents, children identified as Asian, Black, Hispanic, or Native Hawaiian or Pacific Islander were more likely to receive a diagnosis of asthma compared with White children. Black children had the highest odds of diagnosis across all age groups, aligning with national patterns. Compared with earlier studies, our findings reflected a decrease in asthma-related ED visits and PAHs and increases in the use of ICSs and other asthma medications, which may suggest improved adherence to treatment guidelines and better overall management of the condition. These results highlighted how access to low-cost or no-cost care, consistent insurance coverage, and effective prescription practices within the MHS may have helped to improve asthma outcomes. Still, the persistence of racial and ethnic disparities pointed to the need for further action. Efforts to close these gaps should include expanding access to culturally responsive care, increasing availability of specialists, and continuing to assess and improve how care is delivered across the system.

## Notes

Supplement 1. eTable 1. List of *ICD-10* codes used for inclusion and exclusion eTable 2. Characteristics of the full pediatric population eTable 3. Characteristics of pediatric asthma cohort (aged 2-17) during FY 2023

Supplement 2. Data Sharing Statement
