# Supplementary material for: Pediatric Asthma in a Universally Insured Military Population
Source: JAMA Netw Open. 2026 Jan 26;9(1):e2556740. doi: 10.1001/jamanetworkopen.2025.56740 (PMC12836133; doi:10.1001/jamanetworkopen.2025.56740)
Supplement: Supplement 1. — eTable 1. List of ICD-10 codes used for inclusion and exclusion eTable 2. Characteristics of the full pediatric population eTable 3. Characteristics of pediatric asthma cohort (aged 2-17) during FY 2023 [file jamanetwopen-e2556740-s001.pdf]

Supplemental Online Content

Denteh FY, Vaughan W, Banaag A, Wu H, Joseph KA, Koehlmoos TP. Pediatric asthma in a universally insured military population. *JAMA Netw Open*. 2026;9(1):e2556740. doi:10.1001/jamanetworkopen.2025.56740

eTable 1. List of *ICD-10* codes used for inclusion and exclusion

eTable 2. Characteristics of the full pediatric population

eTable 3. Characteristics of pediatric asthma cohort (aged 2-17) during FY 2023

This supplemental material has been provided by the authors to give readers additional information about their work.

© 2026 Denteh FY et al. *JAMA Network Open*.

**eTable 1.** List of *ICD-10* codes used for inclusion and exclusion	
**Code**	**Description**
Included	
J45*	Asthma
Excluded	
E84*	Cystic fibrosis
P270	Wilson-Mikity syndrome
P271	Bronchopulmonary dysplasia originating in the perinatal period
P278	Other chronic respiratory diseases originating in the perinatal period
P279	Unspecified chronic respiratory disease originating in the perinatal period
Q2545	Congenital malformation of the double aortic arch
Q2546	Congenital malformation of the tortuous aortic arch
Q2547	Congenital malformation of the right aortic arch
Q30*	Congenital malformations of the nose
Q31*	Congenital malformations of the larynx
Q32*	Congenital malformations of the trachea and bronchus
Q33*	Congenital malformations of the lung
Q34*	Other congenital malformations of the respiratory system
Q39*	Congenital malformations of the esophagus

© 2026 Denteh FY et al. *JAMA Network Open*.

**eTable 2.** Characteristics of the full pediatric population							
**Characteristic**	**All Races/Ethnicities** n=950,896	**Asian** n=24,150 (2.5)	**Black** n=157,709 (16.6)	**Hispanic** n=166,253 (17.5)	**Native Hawaiian or Pacific Islander** n=33,032 (3.5)	**White** n=526,324 (55.4)	**Other** n=43,428 (4.6)[a]
	n (%)	*n (%)*	*n (%)*	*n (%)*	*n (%)*	*n (%)*	*n (%)*
Age							
2-4	170,915 (18.0)	3,875 (16.0)	29,078 (18.4)	33,463 (20.1)	7,373 (22.3)	90,455 (17.2)	6,671 (15.4)
5-10	351,769 (37.0)	8,607 (35.6)	55,829 (35.4)	63,420 (38.1)	14,017 (42.4)	194,254 (36.9)	15,642 (36.0)
11-17	428,212 (45.0)	11,668 (48.3)	72,802 (46.2)	69,370 (41.7)	11,642 (35.2)	241,615 (45.9)	21,115 (48.6)
Sex							
Male	483,988 (50.9)	12,532 (51.9)	79,580 (50.5)	84,553 (50.9)	16,881 (51.5)	268,372 (51.0)	22,070 (50.8)
Female	466,908 (49.1)	11,618 (48.1)	78,129 (49.5)	81,700 (49.1)	16,151 (48.9)	257,952 (49.0)	21,358 (49.2)
Number of Siblings							
0/Unknown	92,112 (9.7)	2,705 (11.2)	18,283 (11.6)	18,050 (10.9)	3,533 (10.7)	45,503 (8.6)	4,038 (9.3)
1-2	585,810 (61.6)	15,652 (64.8)	86,860 (55.1)	104,073 (62.6)	20,941 (63.4)	331,879 (63.1)	26,405 (60.8)
3+	272,974 (28.7)	5,793 (24.0)	52,566 (33.3)	44,130 (26.5)	8,558 (25.9)	148,942 (28.3)	12,985 (29.9)
Sponsor Marital Status							
Married	867,165 (91.2)	22,647 (93.8)	133,162 (84.4)	151,002 (90.8)	30,314 (91.8)	490,419 (93.2)	39,621 (91.2)
Single	83,731 (8.8)	1,503 (6.2)	24,547 (15.6)	15,251 (9.2)	2,718 (8.2)	35,905 (6.8)	3,807 (8.8)
Sponsor Rank Group[b]							
Junior Enlisted	62,578 (6.6)	2,386 (9.9)	15,008 (9.5)	14,696 (8.8)	1,795 (5.4)	26,461 (5.0)	2,232 (5.1)
Senior Enlisted	648,148 (68.2)	16,833 (69.7)	118,469 (75.1)	124,463 (74.9)	22,188 (67.2)	336,926 (64.0)	29,269 (67.4)
Junior Officer	121,190 (12.7)	2,760 (11.4)	13,103 (8.3)	14,623 (8.8)	5,221 (15.8)	79,439 (15.1)	6,044 (13.9)
Senior Officer	79,764 (8.4)	1,571 (6.5)	5,256 (3.3)	6,094 (3.7)	2,454 (7.4)	60,079 (11.4)	4,310 (9.9)
Healthcare Sector							
Direct Care Only	166,474 (17.5)	5,093 (21.1)	31,387 (19.9)	32,097 (19.3)	7,564 (22.9)	80,988 (15.4)	9,345 (21.5)
Private Sector Only	421,866 (44.4)	11,396 (47.2)	64,343 (40.8)	66,606 (40.1)	9,963 (30.2)	250,159 (47.5)	19,399 (44.7)
Both	362,556 (38.1)	7,661 (31.7)	61,979 (39.3)	67,550 (40.6)	15,505 (46.9)	195,177 (37.1)	14,684 (33.8)
[a]Includes racial identifications of 'Other', 'Unknown', and 'American Indian Alaska Native'.							
[b]Other/Unknown rank groups are excluded							

© 2026 Denteh FY et al. *JAMA Network Open*.

**eTable 3.** Characteristics of pediatric asthma cohort (aged 2-17) during FY 2023[a]							
**Characteristic**	**All Races/Ethnicities** n=31,288 (3.3)	**Asian** n=763 (3.2)	**Black** n=7,848 (5.0)	**Hispanic** n=6,023 (3.6)	**Native Hawaiian or Pacific Islander** n=984 (3.0)	**White** n=14,349 (2.7)	**Other**[b] n=1,321 (3.0)
	n (%)	*n (%)*	*n (%)*	*n (%)*	*n (%)*	*n (%)*	*n (%)*
Age							
2-4	5,080 (16.2)	97 (12.7)	1,197 (15.3)	1,101 (18.3)	192 (19.5)	2,314 (16.1)	179 (13.6)
5-10	13,280 (42.4)	322 (42.2)	3,247 (41.4)	2,782 (46.2)	485 (49.3)	5,893 (41.1)	551 (41.7)
11-17	12,928 (41.3)	344 (45.1)	3,404 (43.4)	2,140 (35.5)	307 (31.2)	6,142 (42.8)	591 (44.7)
Sex							
Male	18,130 (58.0)	471 (61.7)	4,615 (58.8)	3,553 (59.0)	567 (57.6)	8,192 (57.1)	732 (55.4)
Female	13,158 (42.1)	292 (38.3)	3,233 (41.2)	2,470 (41.0)	417 (42.4)	6,157 (42.9)	589 (44.6)
Number of Siblings							
0/Unknown	3,483 (11.1)	103 (13.5)	1,046 (13.3)	736 (12.2)	118 (12.0)	1,343 (9.4)	137 (10.4)
1-2	19,002 (60.7)	498 (65.3)	4,201 (53.5)	3,773 (62.6)	633 (64.3)	9,146 (63.7)	751 (56.9)
3+	8,803 (28.1)	162 (21.2)	2,601 (33.1)	1,514 (25.1)	233 (23.7)	3,860 (26.9)	433 (32.8)
Sponsor Marital Status							
Married	27,985 (89.4)	706 (92.5)	6,416 (81.8)	5,410 (89.8)	876 (89.0)	13,402 (93.4)	1,175 (88.9)
Single	3,303 (10.6)	57 (7.5)	1,432 (18.3)	613 (10.2)	108 (11.0)	947 (6.6)	146 (11.1)
Sponsor Rank Group[c]							
Junior Enlisted	2,261 (7.2)	71 (9.3)	6,92 (8.8)	569 (9.5)	44 (4.5)	824 (5.7)	61 (4.6)
Senior Enlisted	22,482 (71.9)	564 (73.9)	6,053 (77.1)	4,547 (75.5)	708 (72.0)	9,673 (67.4)	937 (70.9)
Junior Officer	3,382 (10.8)	74 (9.7)	575 (7.3)	520 (8.6)	132 (13.4)	1,910 (13.3)	171 (12.9)
Senior Officer	1,900 (6.1)	43 (5.6)	2,41 (3.1)	161 (2.7)	59 (6.0)	1,299 (9.1)	97 (7.3)
Healthcare Sector							
Direct Care Only	1,447 (4.6)	47 (6.2)	390 (5.0)	326 (5.4)	55 (5.6)	531 (3.7)	98 (7.4)
Private Sector Only	14,620 (46.7)	401 (52.6)	3,419 (43.6)	2,645 (43.9)	299 (30.4)	7,236 (50.4)	620 (46.9)
Both	15,221 (48.7)	315 (41.3)	4,039 (51.5)	3,052 (50.7)	630 (64.0)	6,582 (45.9)	603 (45.6)
[a]For the purposes of this study, an asthma diagnosis is defined as having at least one inpatient or two outpatient claims with asthma in the primary or secondary field during fiscal year 2023							
[b]Includes racial identifications of 'Other'. 'Unknown', and 'American Indian Alaska Native'.							
[c]Other/Unknown rank groups are excluded							

© 2026 Denteh FY et al. *JAMA Network Open*.
