# Supplementary material for: Pediatric Asthma in a Universally Insured Military Population
Source: JAMA Netw Open. 2026 Jan 26;9(1):e2556740. doi: 10.1001/jamanetworkopen.2025.56740 (PMC12836133; doi:10.1001/jamanetworkopen.2025.56740)
Supplement: Supplement 2. — Data Sharing Statement [file jamanetwopen-e2556740-s002.pdf]

Data Sharing Statement

Denteh. Pediatric Asthma in a Universally Insured Military Population. *JAMA Netw Open*. Published January 30, 2026. doi:10.1001/jamanetworkopen.2025.56740

Data
Data available: No

Additional Information
Explanation for why data not available: The data that support the findings of this study are available from the United States Defense Health Agency. Restrictions apply to the availability of these data, which were used under Federal Data User Agreements for the current study, and so are not publicly available.
